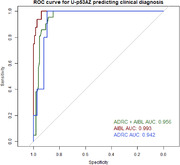# Clinical Utility of U‐p53AZ as a prognostic biomarker for the prediction of progression rate, in individuals with early stages of Alzheimer’s Disease

**DOI:** 10.1002/alz.095720

**Published:** 2025-01-09

**Authors:** Shmuel Agus, Simona Piccirella, Abe Durrant, Garrett B Duncan, Samuel P. Dickson, Suzanne B. Hendrix, Paul Kinnon

**Affiliations:** ^1^ Diadem SpA, Boston, MA USA; ^2^ Diadem SpA, Brescia Italy; ^3^ Pentara Corporation, Salt Lake City, UT USA

## Abstract

**Background:**

Despite recent success in AD clinical trials, many obstacles still prevent effectively halting disease progression. Many suspect that treating subjects earlier in the disease process will be key to providing more effective treatment. Better early diagnostics will aid the design and execution of prevention and early AD studies. Furthermore, early identification of rapid progressors may be valuable in treatment development, since imbalance of rapid progressors can disrupt the proper identification of effective treatment. We present the performance of U‐p53AZ (AZ284) to identify slow progressors and rapid progressors.

**Method:**

A total of 629 participants were randomly selected from the Australian Imaging, Biomarker & Lifestyle Flagship Study of Ageing (AIBL) and the Alzheimer Disease Research Center (ADRC). 172 participants were classified as AD, 303 as cognitively normal, and 154 as MCI or other. U‐p53AZ measurements were taken at baseline and the prognostic value of U‐p53AZ was assessed through multiple analyses. Logistic regression models were fit to predict clinical diagnosis at time points up to 6 years and analyzed through receiver operating characteristic (ROC) analyses. Additionally, U‐p53AZ was used to predict “rapid progressors”. Rapid progressors were defined as patients that progressed in clinical diagnosis to AD within 2 years and as patients which progressed in clinical outcomes using a global statistical test (GST).

**Result:**

U‐p53AZ shows strong predictive performance as a standalone metric throughout both cohorts with an overall AUC of 0.956 when predicting clinical diagnosis at 6 years. Adding demographic variables in the AIBL study raises the AUC over 0.98. For modeling rapid progressors to a clinical diagnosis, U‐p53AZ returns an AUC of 0.887 between both studies. Using a GST of clinical outcomes to define rapid progressors results in an AUC of 0.914. In each case, U‐p53AZ returned a higher AUC compared to PET imaging or CSF.

**Conclusion:**

U‐p53AZ can be used to improve prognostic accuracy of progression to AD over both 2 and 6 years, which will improve screening for AD patients in the clinic and in clinical trials. This prognostic accuracy will help obtain early treatment in those subjects and save resources among those unlikely to develop AD.